# Idiopathic Fulminant Graft Failure Rescued by Urgent ABO-Incompatible Liver Transplantation

**DOI:** 10.1177/15269248211002810

**Published:** 2021-06-03

**Authors:** Fakhar Ali Qazi-Arisar, Raj Uchila, Ashley Limkemann, Zita Galvin, Maryam Monajemzadeh, Mark Cattral, Kathryn Tinckam, Blayne Sayad, Mamatha Bhat

**Affiliations:** 1 Multi Organ Transplant Program, Toronto General Hospital, 7989 University Health Network, Toronto, Ontario, Canada; 2 Division of Gastroenterology and Hepatology, Department of Medicine, University of Toronto, Ontario, Canada; 3 Department of Pathology, 7989 University Health Network, Toronto, Ontario, Canada; 4 Division of Nephrology, Department of Medicine, 7989 University Health Network, Toronto, Ontario, Canada; 5 Department of Laboratory Medicine & Pathobiology, 7989 University Health Network, Toronto, Ontario, Canada

**Keywords:** seventh day syndrome, retransplantation, acute graft failure, graft dysfunction, fatal complication

## Introduction

Seventh-day syndrome (7DS) is a rare but fatal acute complication of liver transplantation. It is characterized by an abrupt rise in liver enzymes and massive hepatocyte necrosis at around a week post-transplant, leading to fulminant hepatic failure in a previously well-functioning graft.^1–3^ We describe a recipient with 7DS, successfully managed with emergent ABO-incompatible (ABOi) retransplant. The patient provided informed consent to publish case.

## Case

A 47-year-old man with well-compensated cirrhosis (Na-MELD 18) and hepatoma secondary to autoimmune hepatitis underwent uncomplicated liver transplant with a brain-dead donor liver. Immunosuppressive induction was methylprednisone. Tacrolimus was introduced on postoperative day (POD) 1.

**Figure 1.**
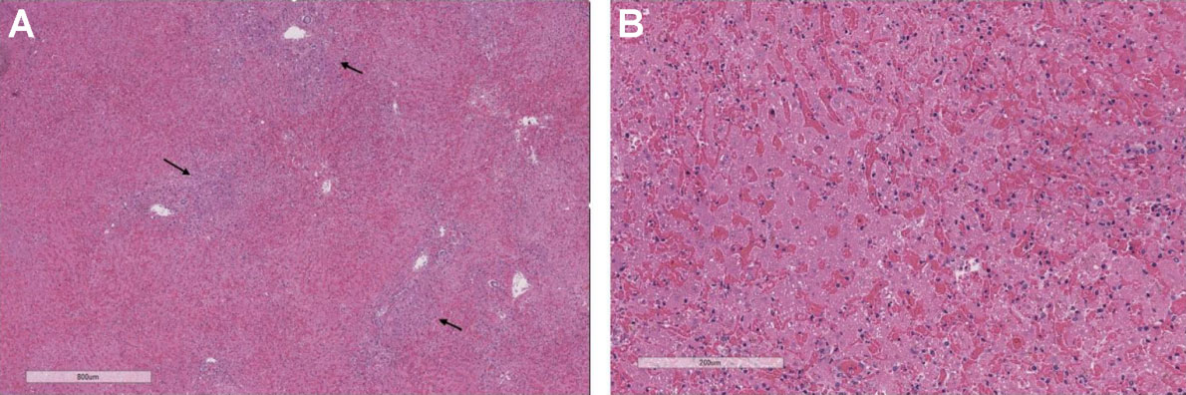
Graft hepatectomy histology from recipient with fulminant transplant liver failure. A, Severe sinusoidal congestion and hemorrhage as well as marked coagulative geographic necrosis. Note the scattered islands of viable hepatocytes at zone 1 (arrows) (hematoxylin and eosin). B, Higher power view of (A), showed severe sinusoidal congestion and coagulative necrosis with almost no inflammation and no obvious apoptotic bodies (hematoxylin and eosin).

He had a routine recovery until POD 6 (with tacrolimus level 9.4 ug/L), when the liver enzymes abruptly increased (AST > ALT) (Table 1). Doppler ultrasound and computerized tomograph with contrast revealed patent vasculature and no clear abnormalities. Viral serologies/PCRs were negative. He developed encephalopathy and acidosis, requiring listing for urgent retransplantation. On POD 7, the enzymes climbed further (Table 1). He was intubated for airway management. Liver biopsy revealed coagulative geographic necrosis with no viral cytopathic effect (Figure 1A and B).

**Table 1.** Laboratory Trend Showing Renal and Liver Functions of Patient Before and After Transplant.

Date & time	INR	Creatinine (µmol/L)	Bilirubin (µmol/L)	AST (IU/L)	ALT (IU/L)	ALP (IU/L)
Pre OP	1.5	49	87	63	49	216
POD-0 4:27	1.6	108	102	754	360	100
POD-4 8:19	1.1	61	34	70	130	79
POD-5 7:54	1.1	58	44	137	177	77
POD-6 8:28	1.4	67	83	865	746	124
POD-6 16:27	1.8		146	3311	2313	137
POD-7 2:29	3.4	66	179	6098	3929	116
POD-7 21:01	8.8	179	278	10385	5699	178
POD-0 5:29	2.3	227	144	6006	2145	126
POD-1 8:03	1.3	85	53	384	327	85
POD-3 7:41	1.0	179	24	102	152	90
POD-10 5:26	1.2	57	14	28	44	57

Abbreviations: INR, International normalized ratio; AST, aspartate aminotransferase; ALT, alanine transaminase; ALP, alkaline phosphatase.

An ABOi offer (donor blood group-B, recipient blood group-O) was accepted due to rapid deterioration and lack of blood group matched offers. His clinical condition continued to worsen, requiring inotropic support, initiation of hemodialysis and operative devascularization of the graft with temporary portocaval shunt. On POD 8, retransplant was performed with simultaneous splenectomy.

Prior to ABOi transplant, 2 sessions of plasmapheresis were completed. Induction therapy was thymoglobulin and methylprednisone. He received 5 sessions of plasmapheresis and IV Immunoglobulin G on alternate days. Surveillance biopsies on PODs 10 and 21 were unremarkable. At 10 months posttransplant, he was doing well with no evidence of rejection and normal graft function.

## Discussion

Seventh-day Syndrome is an uncommon transplant complication, with incidence between 0.5% to 1.68% and male preponderance.^2,3^ There has been no demonstrated association with age, wait time, etiology, MELD score, type of allograft or surgical factors.^2,3^ Massive coagulative necrosis without major inflammation is the most prominent histological feature. The postulated pathogenesis involves an undefined immune response and activation of apoptosis pathways,^3^ as supported by mice models demonstrating increased hepatocyte expression of Fas receptor and induction of hepatocyte apoptosis by systemic injection of monoclonal antibody to Fas receptor.^4^ The mortality rate is above 80%,^2,3^ and retransplantation is the only curative option with 80% 1-year survival.^1^

In North America, ABOi transplant has largely been reserved for patients with fulminant hepatic failure in the absence of a blood group-compatible organ. Intensive desensitization using IVIG, plasmapheresis, and rituximab vs splenectomy has improved graft and patient survival after ABOi transplantation.^5^

Our case describes the rare entity of 7DS, characterized by sudden graft necrosis and failure. This represents a posttransplant emergency, highlighting the need to urgently act upon development of markedly abnormal liver biochemistry and evidence of liver failure, despite initial normal graft function and in the absence of vascular thrombosis. Fortunately, certain mortality was avoided with the successful application of emergent ABOi transplantation.

## Abbreviations

7DS: Seventh Day Syndrome
ABOi: ABO incompatible
ALT: Alanine Transaminase
AST: Aspartate Transaminase
INR: International Normalizing Ratio
IVIG: Intravenous immunoglobulin
Na-MELD: Sodium—Model for End Stage Liver Disease
POD: Post-Operative Day; PCR: Polymerase Chain Reaction.
